# Meditation: Evidence Map of Systematic Reviews

**DOI:** 10.3389/fpubh.2021.742715

**Published:** 2021-12-02

**Authors:** Caio Fábio Schlechta Portella, Ricardo Ghelman, Veronica Abdala, Mariana Cabral Schveitzer, Rui Ferreira Afonso

**Affiliations:** ^1^Brazilian Academic Consortium for Integrative Health, São Paulo, Brazil; ^2^Department of Obstetrics and Gynecology, University of São Paulo, São Paulo, Brazil; ^3^Centro Latino-Americano e do Caribe de Informação em Ciências da Saúde, São Paulo, Brazil; ^4^Preventive Medicine Department, Federal University of São Paulo, São Paulo, Brazil; ^5^Brain Institute, Albert Einstein Israelite Hospital, São Paulo, Brazil

**Keywords:** meditation, evidence map, systematic review, complementary and alternative medicine, mindfulness

## Abstract

**Study Basis:** This evidence map presents a summary of studies that addressed the effects of meditation on various clinical and health conditions. Meditation is a contemplative practice that has been used for the promotion of health, and the treatment of different conditions.

**Method:** The study is based on the search of four electronic databases for the period 1994-November 2019 and includes systematic reviews, meta-analyses, meta-syntheses, and integrative reviews. 3iE evidence gap map was the methodology of choice, and AMSTAR 2 was used for the analyses. Tableau was used to graphically display the confidence level, number of reviews, health outcomes, and intervention effects.

**Results:** This map encompasses 191 studies, with Mindfulness being the key word that retrieved the highest number of results. Several meditation techniques were evaluated in different contexts, and the confidence levels of 22 studies were high, 84 were moderate, and 82 were low. Two 2 meta-syntheses and 1 integrative review were also included. Most of the studies reported positive effects and a beneficial potential of the practice of meditation. Health outcomes were divided into five groups out of which mental health and vitality, and well-being and quality of life stood out with the largest number of studies.

**Conclusions:** Meditation has been applied in different areas. This Evidence Map intends to be an easy visual tool to access valuable evidence-based information on this complementary therapy for patients, health professionals, and managers.

## Background

The World Health Organization (WHO) has encouraged and strengthened the inclusion, recognition, and use of Integrative and Complementary Health Practices (ICPs) in national health systems at all levels: Primary Care, Specialized Care, and Hospital Care, through the recommendations of the WHO Strategy on Traditional Medicine 2014–2023 (1).

Since 2018, meditation is one of the 29 practices that have been included in the Brazilian Unified Health System (2). Meditation is a contemplative practice, related to the development of human experiences and widely used in different traditions. It originated centuries ago and is currently studied for its effects on health. Meditation is a generic term that many practices fit into. Therefore, some authors propose a general definition of meditation. Cardoso et al. (3) define it as: “Meditation is a procedure which should encompass a specific technique, clearly defined, involving muscle relaxation in some point of the process and “logic relaxation”; it is a self-induced state, using a self-focus skill (coined “anchor”). Within the various schools of meditation, Mindfulness has a prominent place due to the considerable number of works found on this map. Some authors define Mindfulness as: “paying attention in a particular way: on purpose, in the present moment, non-judgmentally” (4) or “mindfulness meditation involves the self-regulation of attention and involves adopting a particular orientation toward one's experiences in the present moment” (5). Given the considerable number of meditation schools, we chose to add studies that self-identified as meditation.

Due to the recent expansion of the National Policy for Integrative and Complementary Practices (PNPIC), the Brazilian Ministry of Health has signed a partnership with the Latin American and Caribbean Center for Health Sciences Information (BIREME-PAHO-WHO) and the Brazilian Academic Consortium for Integrative Health (CABSIN) to develop Evidence Maps on Integrative and Traditional Health Practices (6), including this one on meditation. The objective of this Evidence Map is to concisely describe different meditation interventions and their related health outcomes.

According to Johnson (7), meditation is a practice that may have arisen simultaneously with the domestication of fire. The author explains that the first meditative experiences may have taken place while our ancestors contemplated the fire flames. In the past, meditation was considered a practice within the spiritual realm; however, the recent increase in the number of practitioners may derive from its positive effects on health (8, 9).

The first robust evidence in the health field can be found in the late 60's. In 1970 (10), Wallace used EEG and other physiological measures to monitor the meditative state. The results of this study pointed to the surge of theta waves, a frequency decrease, and an increase in the amplitude of the alpha waves. Thus, the author characterized meditation as a hypo metabolic state with a predominant expression of the parasympathetic nervous system and a reduction of the sympathetic tonus.

Since then, the number and quality of studies on meditation has increased, becoming an integrative practice that has gained a growing interest among health professionals.

About 19% of the non-institutionalized adult population in the USA practices some kind of meditation (11). A higher rate of adoption has been observed among women, and the more educated and younger population, and people with chronic diseases as anxiety, depression, pain, and sleep problems. The high cost of conventional medical treatment is also one of the reasons for the adoption of this practice. The most vulnerable population, with worse health conditions, is less likely to practice meditation (12).

The interest in Complementary and Alternative Medicine, classified by WHO within a broader scope as Traditional, Complementary, and Integrative Medicines (MTCI), has grown not only within the general population, but also among health professionals. In general, about 80% of complementary therapies have been considered as an option of treatment during the patient-doctor discussions (13). Despite the moderate level of knowledge that doctors have about meditation, they find it beneficial and prescribe it as a form of treatment (14). This acceptance of the meditation practice may derive from the increasing number of courses on MTCI offered as part of the medical curriculum; however, little scientific evidence is offered on this regard (15).

To bridge this gap, this study intends to systematize research results on the prescription and adoption of meditation in a clear and concise map to boost confidence and inform decisions taken by health professionals and public administrators.

## Method

This Evidence Map includes integrative and systematic reviews, meta-analysis, and meta-synthesis, and summarizes meditation related health interventions and outcomes. Systematic reviews were performed to obtain a reliable summary of the best evidence available in this area. The analysis was carried out following the PRISMA guidelines (16) and the International Initiative for Impact Evaluation (3iE) Evidence Gap Map methodology (17). An advisory panel of librarians, practitioners, policy makers, content experts, and researchers contributed to the production of this evidence map.

## Data Sources

Papers published in English, Spanish, and Portuguese were selected in searches conducted on PUBMED, BVS, and EMBASE databases from 1994 to November 2019. Experts on the topic were consulted and the search strategy was jointly developed with BIREME.

List of the words used in the search in Table 1 terms searched for the meditation evidence map.

**Table 1:** Terms searched for the meditation evidence map.

**Database**	**Searched words**
BVS	((MH:Meditation OR ti:(Meditation OR Meditacao OR Meditacion OR Mindfulness OR “Atención Plena OR “Atenção Plena” OR “Consciência Plena” OR “Terapia de relaxamento”)) AND (therap* OR medicinal OR medical OR terap* OR treatment* OR tratamento* OR eficacia OR efficacy OR clinic* OR effect* OR MH:”/uso terapeutico” OR MH:”/terapia” OR “uso clinic” OR “uso terapeutico” OR “Therapeutic use” OR “Clinical use” OR “aplicação clínica” OR “aplicación clinica” OR “aplicação terapêutica”)) andnot “MEDLINE”
PUBMED	((((MH:Meditation OR TI:Meditation OR TI:Meditação OR TI:Meditacion OR TI:Mindfulness OR Cogitat* OR Pranayam* OR kapalabhati OR TI:zen OR TI:transcendental OR “M-Sidhi” OR mahayana OR hiniyana OR theravada* OR vajrayana OR vipassana OR vipashyana OR dhyana OR dyana OR dharana OR zazen OR kinemantra OR TI:KM OR mantra OR mantras OR samadhi OR samatha OR pratyahara OR purusha OR prakruti OR Hesychasm OR “lectio divina” OR bonadona OR “Anapana Sati” OR anapanasati OR “kabat-zinn” OR “sudarshan kriya” OR TI:raja OR ratha) OR ((“Atención Plena” OR “Atenção Plena” OR “Consciência Plena” OR “Terapia de relaxamento” OR “mind-body and relaxation techniques” OR “mindbody relations” OR “open awareness” OR “focused awareness” OR “relaxation response” OR “progressive muscle relaxation” OR “progressive relaxation” OR “forced nostril breathing” OR “Uninostril breathing” OR “unilateral breathing”) AND NOT (qigong OR “qi gong” OR “ch'i kung” OR “Tae Eul Ju” OR “tai ji” OR “tai chi” OR “tai ji” OR “Taijiquan” OR Khundalini OR Kundalini OR TI:yoga))) AND (therap* OR medicinal OR medical OR terap* OR treatment* OR tratamento* OR eficacia OR efficacy OR clinic* OR effect* OR MH:”/usoterapeutico” OR MH:”/terapia” OR “usoclinico” OR “usoterapeutico” OR “Therapeutic use” OR “Clinical use” OR “aplicaçãoclinica” OR “application clinica” OR “aplicaçãoterapeutica”)) AND NOT DB:”MEDLINE”)
EMBASE	#38 AND #39 AND ('therapeutic use' OR 'clinical use' OR 'clinical') AND [embase]/lim NOT ([embase]/lim AND [medline]/lim) AND ('meta-analysis': de OR 'systematic review':de) #38 AND #39 #39 #36 OR #37 #38 therap*:ti OR medicinal:ti OR medical:ti OR treatment*:ti OR efficacy:ti OR clinic*:ti OR effect*:ti OR 'therapy'/exp OR 'therapeutic use' OR 'clinical use' #37 'meditation'/mj OR mindfulness:ti OR meditation:ti OR cogitat* OR pranayam* OR kapalabhati OR zen:ti OR transcendental:ti OR 'm sidhi' OR mahayana OR hiniyana OR theravada* OR vajrayana OR vipassana OR vipashyana OR dhyana OR dyana OR dharana OR zazen OR kinemantra OR km:ti OR mantra OR mantras OR samadhi OR samatha OR pratyahara OR purusha OR prakruti OR hesychasm OR 'lectio divina' OR bonadona OR 'anapana sati' OR anapanasati OR 'kabat-zinn' OR 'sudarshan kriya' OR raja:ti OR ratha #36 ('mind-body and relaxation techniques' OR 'mindbody relations' OR 'open awareness' OR 'focused awareness' OR 'relaxation response' OR 'progressive muscle relaxation' OR 'progressive relaxation' OR 'forced nostril breathing' OR 'uninostril breathing' OR 'unilateral breathing') NOT (qigong OR 'tai chi' OR 'tai ji' OR 'taijiquan' OR khundalini OR kundalini OR yoga:ti OR 'yoga'/mj)

### Inclusion Criteria

Systematic reviews/meta-analysis, and meta-syntheses on meditation with adequate description of health outcomes were eligible for inclusion in the Evidence Map. An integrative review study was considered as highly relevant and was also included. The selection of systematic reviews, meta-analysis, meta-synthesis, and integrative reviews was done based on the studies that self-identified as such. Participants of all ages, regardless of their health condition, were also eligible for inclusion as well as interventions of any kind, follow-up, and duration. Studies that did not focus on meditation and/or related health outcomes were excluded.

### Procedures

**Figure 1:**
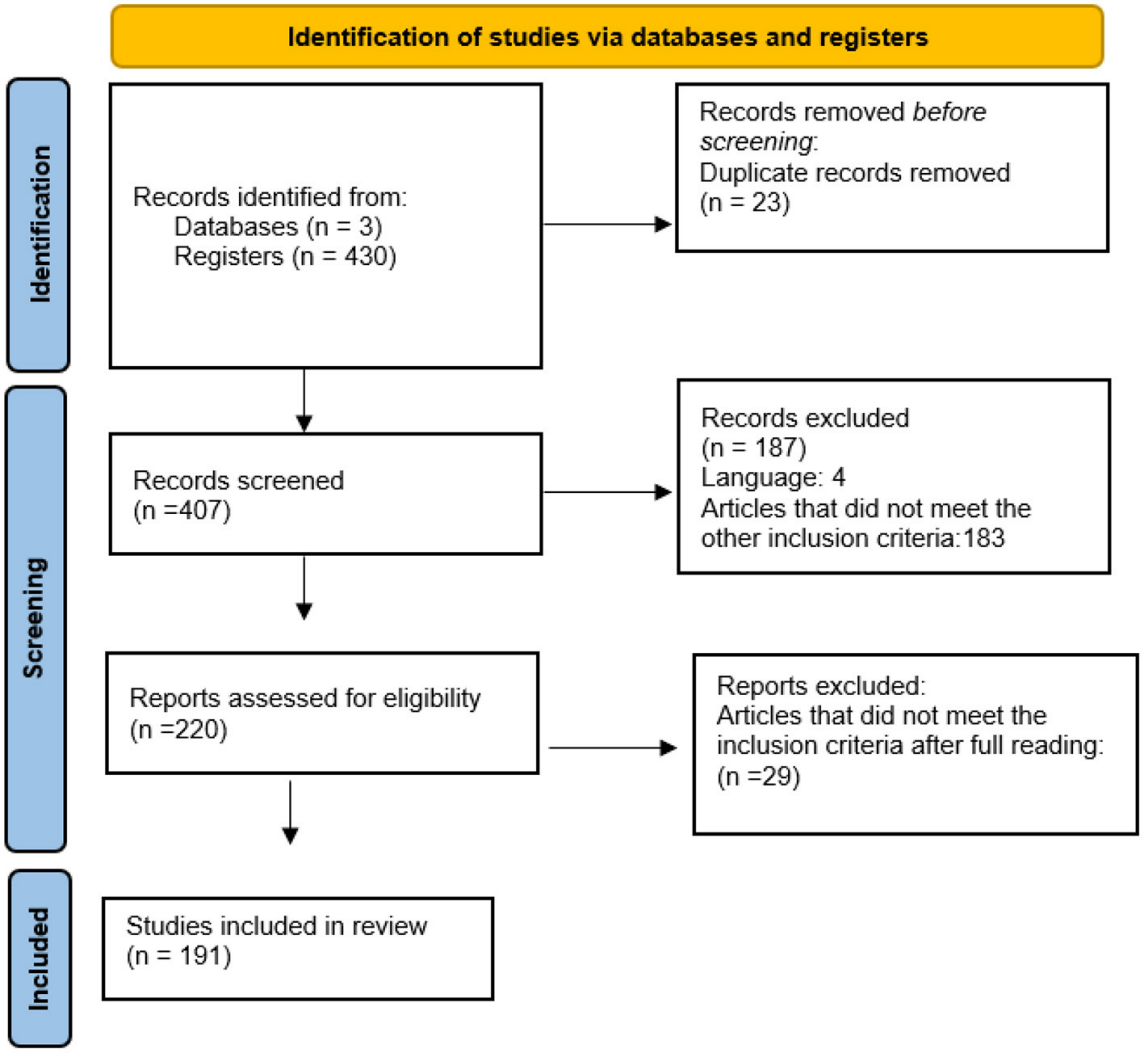
Flowchart of analyzed studies.

**Figure 2:**
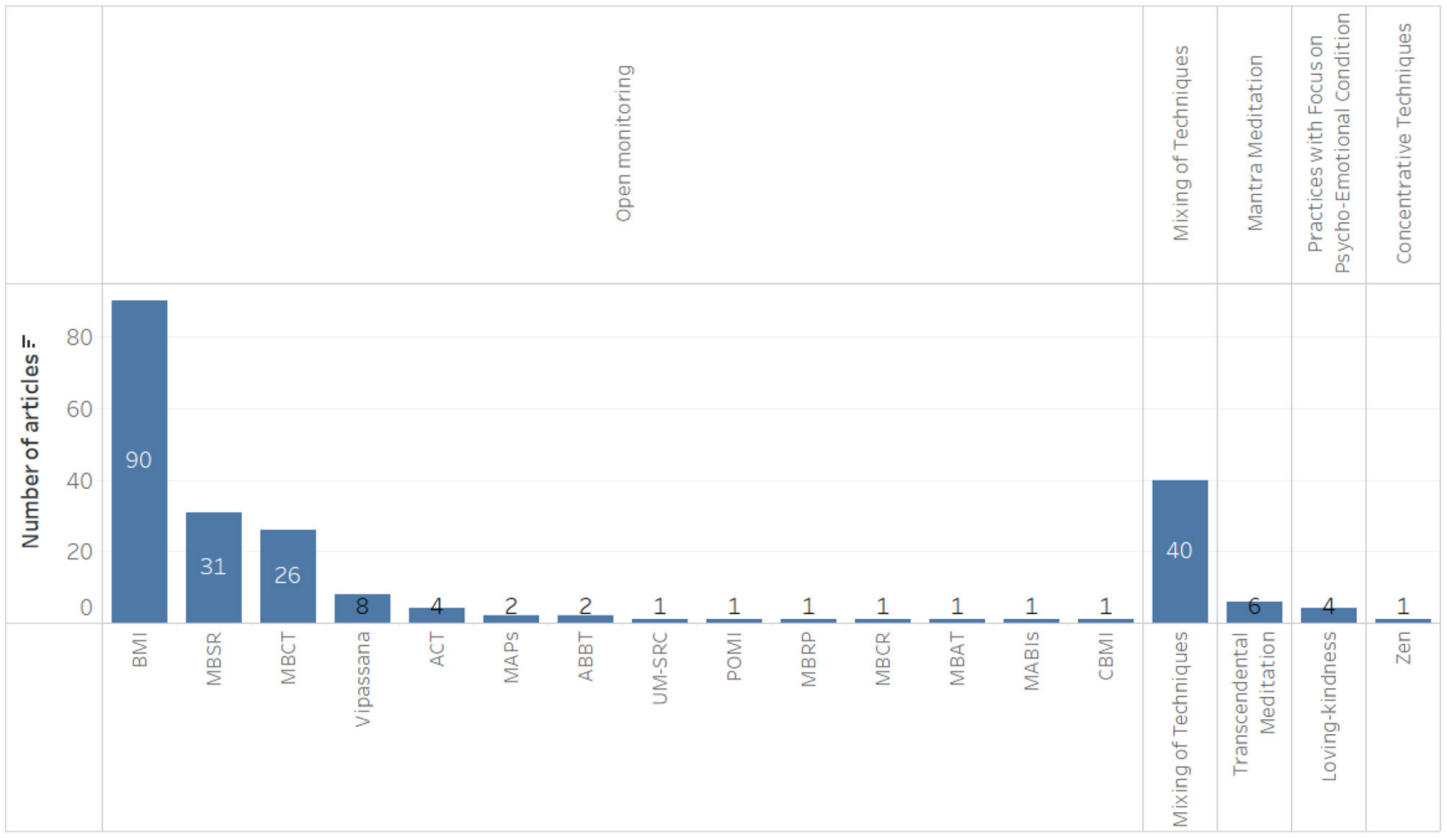
Types of meditation practices identified.

Screening and blinding procedures were carried out by two independent reviewers using Rayyan software (18). Citations deemed as potentially relevant by at least one reviewer and unclear citations were verified in their full text. Full-text publications were selected according to the inclusion criteria agreed by the two independent reviewers; disagreements were resolved by discussion (Figure 1). AMSTAR 2 (19) (Figure 2) was used to analyze the quality and methodological rigor of the studies included.

### Synthesis of the Data

A spreadsheet with various descriptors was developed to synthesize the findings. This matrix included: Full text citation; Interventions; Outcomes group; Outcomes; Effect; Population; Database ID; Country of focus; Country of publication; Year of publication; Type of review; Review design; Study design; Confidence level. The Evidence Map was organized considering the outcomes, effects, and confidence level of the studies included.

## Results

Of 407 citations identified, 191 studies met the inclusion criteria. A total of 507 different outcomes were identified, with it being possible to find more than one per study, i.e., a single article may have information related to two outcome groups, several types of meditation, and different populations. One hundred and ten systematic reviews with meta-analysis, 78 systematic reviews, two meta-syntheses, and one integrative review were included. Meditation types based on open state practices accounted for the highest number of results (390 results), followed by mixture of techniques (93 results), mantra-focused practices (15 results), state-focused practices (8 results), and focused mindfulness practices (1 result). According to AMSTAR2 classification, most studies showed a moderate level of confidence (*n* = 84 studies), 82 studies had a low confidence level, and 22 studies had a high confidence level. The two meta-syntheses and the integrative review could not be evaluated by this tool (19).

### Population

Most of the studies found in the search addressed outcomes in the adult population with some diagnosed conditions, with 114 on patients with mental disorders, 45 on patients with cancer, and 36 on patients with chronic pain. The search also identified 36 studies conducted exclusively with children or adolescents, and 35 that only included women.

### Countries

The United States was the country of focus where the highest number of studies were carried out (61 studies), followed by Canada (30 studies), United Kingdom (28 studies), and Australia (23 studies). One hundred and twenty one studies did not report the country of focus.

### Outcomes and Effects

**Figure 3:**
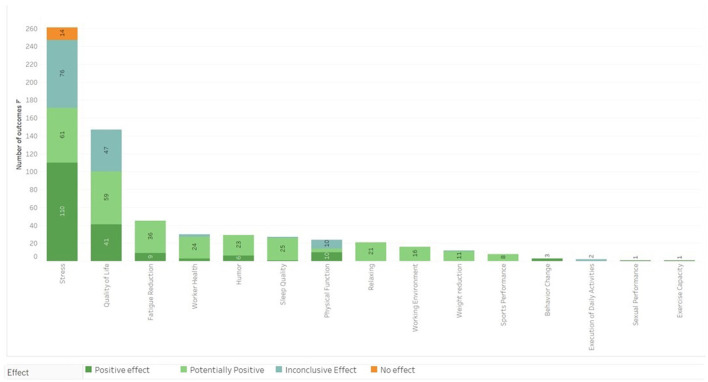
Main outcomes categorized by results and confidence level.

**Figure 4:**
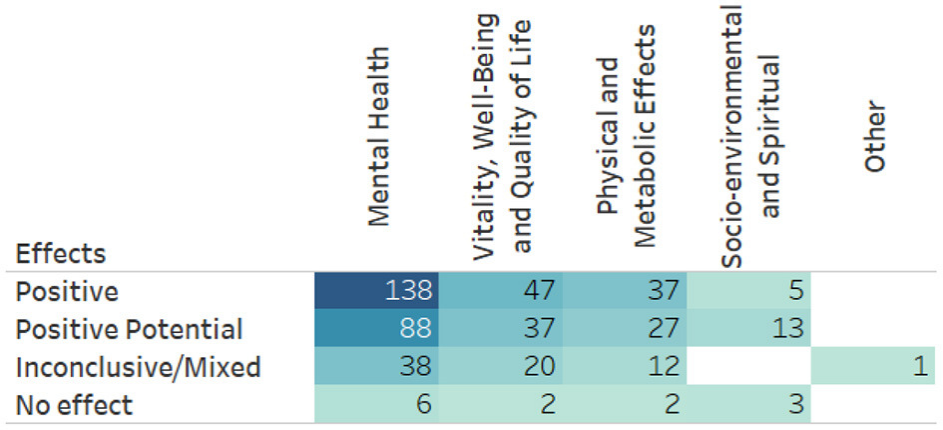
Results of meditation effects by category in the research included in the Evidence Map.

It was observed that meditation has been used as an intervention in diverse health conditions. Within the 191 studies retrieved, effects were classified into five categories of health outcomes and considered as: negative (no results); positive (246 results); potentially positive (174 results); inconclusive/mixed (74 results); and no effect (13 results), reaching a total of 507 diverse effects, as shown in Figure 3. Figure 4 depicts the results, which are divided into five major groups.

### Mental Health

**Figure 5:**
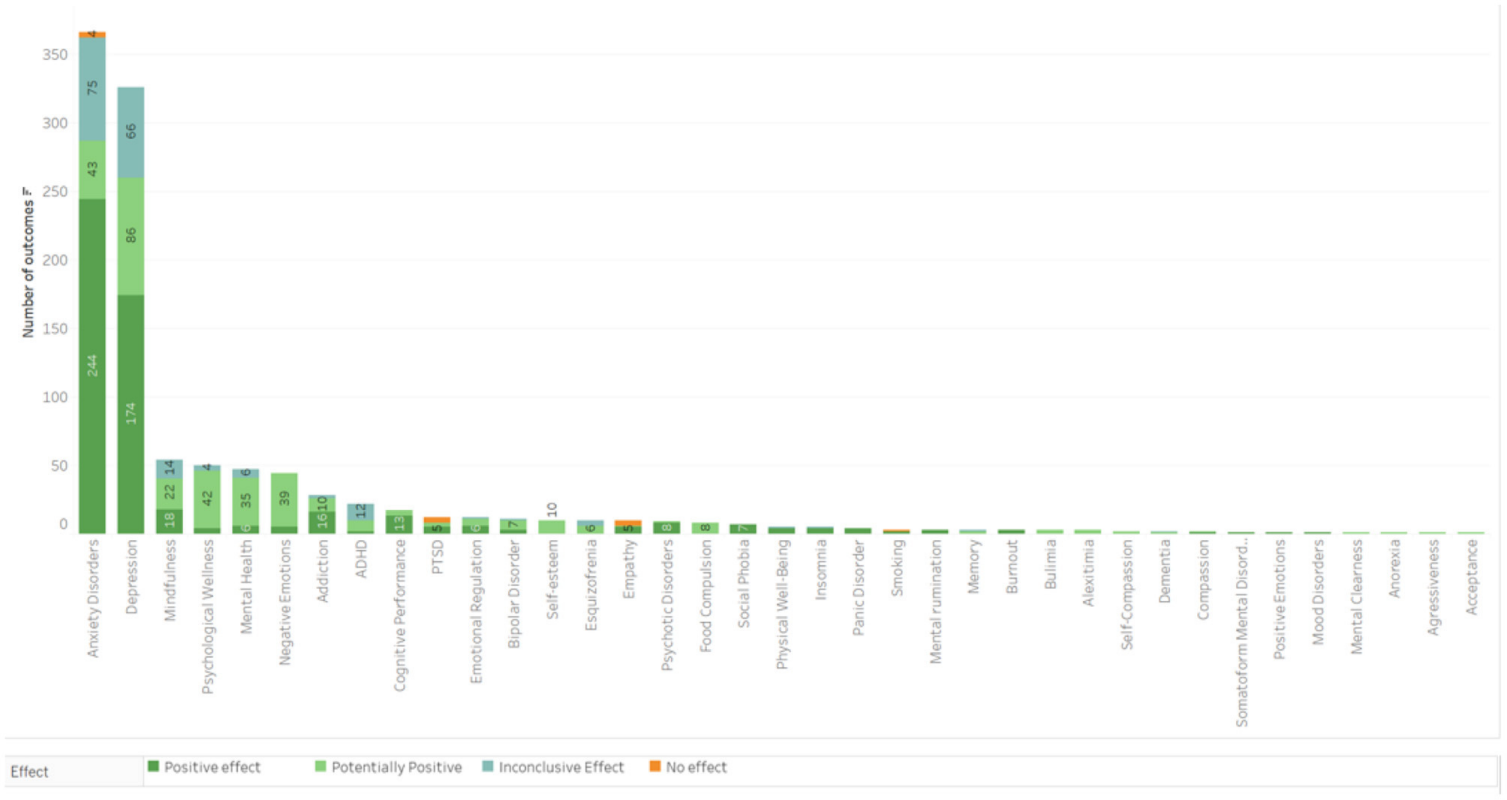
Main outcomes in the Mental Health category.

The Mental Health category included 270 results involving the practice of meditation (Figure 5) among which 138 reported positive effects, 88 reported potentially positive effects, 38 reported inconclusive/mixed effects, and 6 did not describe the effects. The highest number of results was found in the treatment of anxiety, depression, and stress, followed by those pointing out the improvement in levels of mindfulness (Figure 5). Regarding the population, in 97 results the inclusion criterion was patients with some diagnosis of mental disorder, 24 results referred to the effect of meditation practices exclusively in children and adolescents, and in 20 results the inclusion criterion was cancer patients. Results whose inclusion criterion was exclusively women had 18 occurrences. Open State practices were the most prevalent, represented in the treatment of 34 different categories among which Mindfulness-Based Interventions were the most common, followed by Mixed Techniques, adopted in 16 categories, mantra-focused practices, adopted in six categories and focused attention adopted for the treatment of four different categories.

### Physical and Metabolic Effects

**Figure 6:**
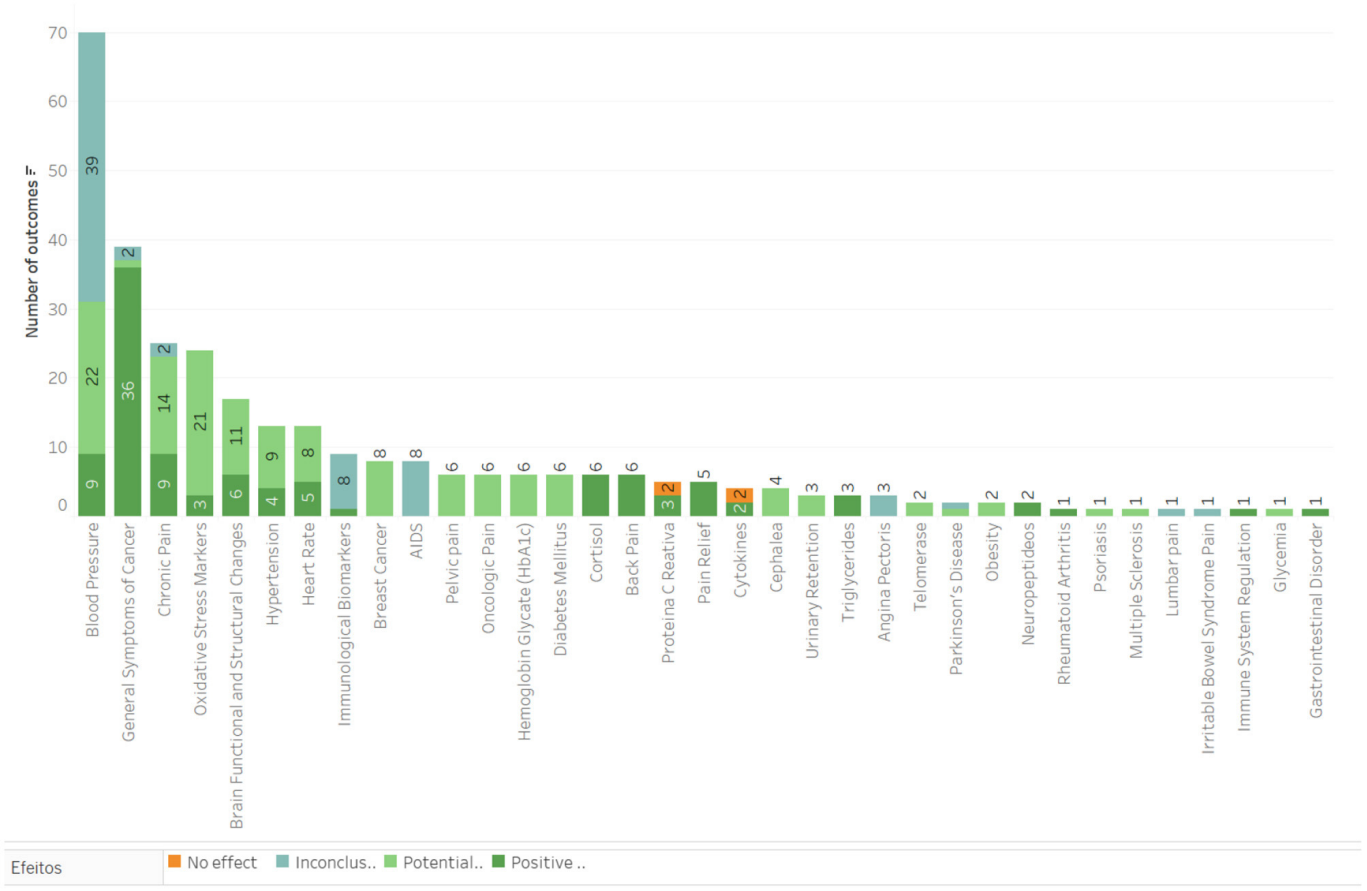
Main outcomes in the category of Physical and metabolic effects.

The group including Physical and Metabolic outcomes presents a total of 87 results. The effect of meditation was positive in 41 results, potentially positive in 29 results, inconclusive in 15 results, and with no effects in two results. The most common outcomes were improvements regarding high blood pressure, general cancer symptoms, and chronic pain (Figure 6). Regarding the population, the inclusion criteria for cancer patients showed effects in 16 outcome categories, cardiovascular/vascular disease 15, and chronic pain 14. Mindfulness-Based Interventions were applied in 20 categories, Mix of Techniques in 11, and Mindfulness-Based Stress Reduction in 14 outcome categories. Most of the studies in this group were classified as having a moderate level of confidence.

### Vitality, Well-Being, and Quality of Life

**Figure 7:**
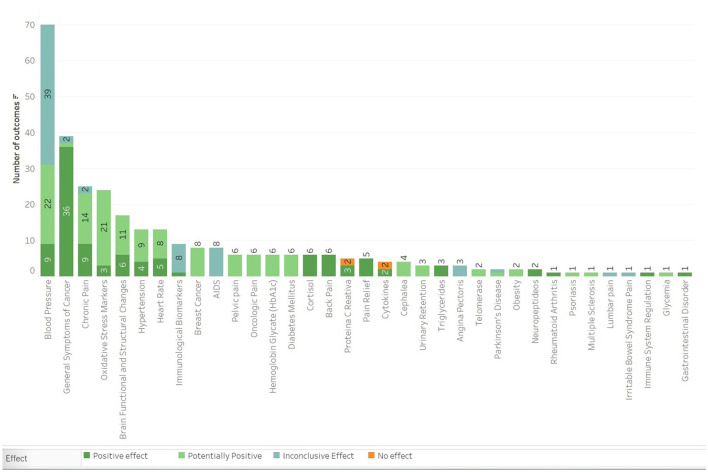
Main outcomes in the Vitality, well-being, and quality of life category.

The vitality, well-being and quality of life group obtained 106 results for the practice of meditation as can be seen in Figure 7, out of which 47 were positive, 37 potentially positive, 20 inconclusive and two results with no effects. The most common outcomes were decreasing stress, higher quality of life and better worker health with 39, 24, and 4 results, respectively. Most of the results obtained in this group (62 results) included a population with some health disorder diagnosed, plus 11 results were obtained using the criteria of workers.

## Discussion

The number of meditation practitioners has grown in recent years. One of the main reasons is the acknowledgment of the positive effects that meditation exerts on health in general (8, 9). However, healthcare courses themselves offer few disciplines and little scientific evidence on the MTCI (15, 20), that is why studies of this nature are necessary, such as systematic reviews, meta-analyses, and evidence mapping.

A survey carried out by Andrade et al. (21), found out that ~30% of the population in a large city like São Paulo suffer some sort of mental disorder, the most prevalent being depression and anxiety. Our survey mostly researched population with some mental disorder. The high prevalence of psychiatric disorders around the world is one of the reasons why a large number of studies on meditation were carried out within the Mental Health outcome group. Within a US sample, anxiety, stress, and depression were the health issues most associated with the search for meditation. Most respondents recognized that meditation improves mental health (22).

In the map, depression, and anxiety were the disorders with the highest number of results, with 71 and 67 results, respectively. On depression, there were 11 reviews with a high confidence level of which only one effect was inconclusive, one potential positive and 9 had positive effects related to meditation. All articles with a high confidence level were systematic review studies with meta-analysis (23, 24) reporting positive effects for reducing depressive symptoms in patients with mental disorders.

Goldberg et al. (24) also conclude that meditation has similar results to some standard treatments such as psychotherapy. Another study showed that a meditation program reduced relapse in patients with depression (25), that is, since recurrent depression is more difficult to be treated and causes high costs for society (26, 27), the MBCT program can be considered an efficient treatment, as observed in NHS experiences in England (28) and confirmed by several studies present in this map (29–31).

Mental health costs in China increased more than 3-fold from 2005 to 2013 and could have reached much higher values if all patients with mental illness received adequate professional treatment. Anxiety is the most prevalent mental disorder, affecting up to a third of the population at some point in life (21, 32, 33) with a high economic and social impact (34). Of all the studies included in this map, most have addressed anxiety. Of the 67 results for Anxiety Disorders, the vast majority, 56 results, were positive or potentially positive, that is, meditation can be used as one of the forms of integrative and complementary treatment for anxiety disorders, as shown in Montero-Marin et al. (35). In other studies, anxiety was assessed in patients with other comorbidities, such as cancer (36–39). The prevalence of anxiety in cancer patients is high (40, 41) and meditation can help patients to reduce anxiety symptoms as stated in the meta-analysis carried out by Zhang et al. (42) which included RCT studies of patients diagnosed with cancer.

In the dimension of health promotion and vitality, 7 moderate quality reviews highlight relevant effects on quality of life (37, 39, 43–48) obtained through Mindfulness interventions. These practices can even be done online (49). The fact that meditation is a potentially self-applicable practice that can be taught from a distance is especially important in periods of social isolation such as the one imposed by the covid-19 (50) pandemic.

The use of meditation as a health promotion resource is growing, with positive outcomes in terms of cognitive performance (51–53) and sexual performance (54), for example. Also noteworthy is the development of mindfulness skills (39, 48, 55–57), compassion (58), empathy (58), and positive emotions (59).

Integrative resources have been increasingly used in the context of health promotion (60). Sarris et al. emphasize the importance of incorporating practices that support lifestyle change based on scientific evidence to support health protocols and policies (60).

Along the same lines, other authors propose broad intervention programs involving lifestyle changes based on meditation (61). Thus, the interdependence between psychological, physical, and human development factors for an approach involving meditation has become relevant (62).

Among the interventions of the Complementary and Integrative Traditional Medicines (MTCI), meditation is the most comprehensive in terms of results and variety of effects. Among the systematic review studies included in the evidence maps presented in the BVS/MTCI, the meditation map is the largest of them, indirectly showing the substantial number of studies published on the subject in recent decades. Meditation techniques can be used by children and adolescents (63), pregnant women (64), the elderly (65), health professionals, and caregivers (66, 67), in addition to people with chronic diseases (68), having a great potential from the point of view of public health, given its impact on physical and metabolic health conditions (63). In cancer patients, it is possible to highlight the increase in variables such as physical well-being (69, 70) and psychological well-being through meditation.

On the physical and metabolic effects of meditation, the literature has described several relevant factors. The ability to promote neural plasticity of meditation techniques is very relevant and has important physical and metabolic impacts (71). Black and Slavich describe possible effects of meditation to improve the immune system (72). The literature also speaks of inflammatory and metabolic markers (73) that are altered with meditative practice.

In this perspective, perhaps interventions such as meditation are part of a plausible solution to deal with the challenges related to quaternary prevention in collective health (74, 75) mainly due to the need to deal with chronic diseases in a preventive and comprehensive approach, considering lifestyle changes in addition to symptom-focused attention.

The creation and publication of evidence maps consists of graphically representing the best evidence found, analyzed and categorized, with the corresponding bibliographic records and full texts (when available) of the studies, in order to facilitate access to information for all interested parties (50).

As it is not the objective of an evidence map, the calculation of the effect sizes of a meta-analysis was not performed, nor was the risk of bias assessments performed, but we tried to overcome these limitations by assessing the quality of the included systematic reviews by applying AMSTAR 2.

The outcomes and effects information presented in this Meditation Evidence Map will further advance the evidence-based knowledge of MTCI as proposed by PNPIC in Brazil and promoted by WHO 2014–2024 and BIREME.

## Conclusions

Meditation practices can be developed by healthy individuals or individuals who have different health conditions, from chronic diseases to different mental health disorders, with positive and potentially positive effects on various low-risk health outcomes.

The practice of meditation can be a safe and effective strategy for implementing programs in the field of collective health. The possibility of applying it in groups facilitates access and reduces costs in relation to drug interventions, for example in mild cases of depression and anxiety, being a potential resource to avoid chronicity and the negative impact of stress on health.

## Data Availability Statement

Publicly available datasets were analyzed in this study. This data can be found here: https://mtci.bvsalud.org/pt/mapas-de-evidencia-2/.

## Author Contributions

CS and RA did the selection of articles and quality assessment, analysis of results, and writing of the discussion. VA designed the search strategies and accompanied the filtering of results by reviewers on the RAYYAN platform, collaborated in the data analysis, methodological design of the evidence maps, and writing of the manuscript. MS and RG collaborated in the methodological design of the evidence maps, data analysis, and writing of the manuscript and conclusions. All authors contributed to the article and approved the submitted version.

## Funding

The study was part of a project funded by the Ministry of Health of Brazil in partnership with the Latin American and Caribbean Center on Health Sciences Information BIREME-PAHO-WHO and the Brazilian Academic Consortium of Integrative Health to develop Evidence Maps in Integrative and Complementary Practices, including meditation. The Brazilian Ministry of Health funded the project and BIREME's research partners conducted the study and performed the collection; management; analysis; interpretation of data; preparation, review, and decision to submit the manuscript for publication.

## Author Disclaimer

The results and conclusions of this publication are the responsibility of the authors responsible for its content; the findings and conclusions do not necessarily represent the views of the Ministry of Health of Brazil and BIREME-PAHO-WHO.

## Conflict of Interest

The authors declare that the research was conducted in the absence of any commercial or financial relationships that could be construed as a potential conflict of interest.

## Publisher's Note

All claims expressed in this article are solely those of the authors and do not necessarily represent those of their affiliated organizations, or those of the publisher, the editors and the reviewers. Any product that may be evaluated in this article, or claim that may be made by its manufacturer, is not guaranteed or endorsed by the publisher.
